# Systematic review and meta-analysis of ropivacaine use in laparoscopic hysterectomy

**DOI:** 10.4274/tjod.galenos.2021.06606

**Published:** 2021-03-12

**Authors:** Greg J Marchand, Ali Azadi, Katelyn Sainz, Sienna Anderson, Stacy Ruther, Kelly Ware, Sophia Hopewell, Giovanna Brazil, Alexa King, Kaitlynne Cieminski, Allison Steele, Jennifer Love

**Affiliations:** 1 Marchand Institute for Minimally Invasive Surgery, Mesa, Arizona, USA; 2 Star Urogynecology, Peoria, Arizona, USA; 3 Washington University of Health and Science, San Pedro, Belize; 4 International University of Health Sciences, Basseterre, St. Kitts; 5 Midwestern University Faculty of Medicine, Glendale, Arizona, USA

**Keywords:** Laparoscopy, ropivacaine, hysterectomy, ERAS, MIGS

## Abstract

To assess the efficacy of all forms of ropivacaine administration for the management of pain and opioid use, specifically in patients undergoing laparoscopic hysterectomy. We searched PubMed, Cochrane CENTRAL, Web of Science, and SCOPUS for relevant clinical trials matching our eligibility criteria. Outcomes of interest included: Pain intensity (measured either by visual analog scale score or by numerical rating scale score), QoR-40 score (Overall quality of recovery tool, designed to measure physical comfort, physical independence, pain, emotional status, and need for support), and the need for opioid rescue. We performed the analysis under the fixed-effects model for homogeneous data and random-effects model for heterogeneous data. Most heterogeneous data were solved by the leave-one-out method, in cases where this was not successful, we then proceeded to conduct at least one subgroup meta-analysis in an attempt to solve heterogeneity. We assessed the risk of bias using Cochrane’s risk of bias tool. A total of five clinical trials were included. Regarding the pain score, there was no significant difference between either group [standardized mean difference=-0.17, 95% confidence interval (CI): (-0.56, 0.23); p=0.41]. The analysis of the overall RoQ40 scores favored the ropivacaine group over the control group significantly [mean difference (MD)=17.68, 95% CI: (1.48, 33.87); p<0.001]. Regarding the use of opioids, the analysis revealed no significant difference between either group [MD=-2.57, 95% CI: (-6.62, 1.49); p=0.21]. Ropivacaine administration by any method does not seem to be effective in reducing pain or reducing the need for opioid use after laparoscopic hysterectomy procedures; however, the administration did show a significant improvement in the patient’s “overall quality of recovery,” as measured using the QoR-40 tool.

## Introduction

Hysterectomy is the most common gynecologic surgical procedure undergone by women in the United States, with over 600,000 performed annually^(1)^. In gynecologic surgery, we have seen a persistent increase in the rate of hysterectomies performed via laparoscopic techniques over time^(2,3)^. About 30% of hysterectomies are performed using minimally invasive laparoscopic techniques^(4)^. Laparoscopic surgeries have many benefits over abdominal approaches: they ensure faster recovery, fewer complications, less pain, and shorter hospital stay^(5)^. Many trials have been conducted to develop strategies to facilitate laparoscopic hysterectomy as an outpatient procedure when feasible^(6)^. However, pain control is still a major problem in postoperative care. Obtaining adequate postoperative analgesia can increase the advantages of the laparoscopic approach over abdominal surgery, and at least in theory, a painless recovery from laparoscopic surgery is possible. For this reason, many trials have been performed to find the best strategies to relieve pain after laparoscopic surgery^(7)^.

Patients undergoing laparoscopic hysterectomy have substantial pain and may require a large dose of opioids in the first 24 hours after the procedure^(8)^. Administration of opioids has many adverse effects such as nausea, vomiting, constipation, and respiratory depression^(9)^. The opioid-related adverse effects may impair the postoperative quality of recovery of patients undergoing this procedure. Administration of local analgesics either intraperitoneally or through the transversus abdominis plane (TAP) may reduce the total need for opioids in the first 24 h postoperatively^(10)^.

Ropivacaine is a long-acting amino amide local anesthetic agent with a duration of action that may extend to 8 hours^(11)^. Ropivacaine is commonly used for nerve block and intraperitoneal use. It produces its analgesic effect via reversible inhibition of sodium ion influx in nerve fibers^(12)^. Ropivacaine is less lipophilic than other local analgesic agents and less likely to penetrate large myelinated motor fibers, resulting in a relatively reduced motor blockade. The reduced lipophilicity is associated with less undesirable central nervous system toxicity and cardiotoxicity. For this reason, it is suitable for immediate pain control after uterine surgeries^(13,14)^.

TAP block is widely used as a pain management approach after various abdominal surgical procedures^(15,16)^. The TAP block consists of an injection of a local anesthetic agent between the internal oblique abdominal muscle and the transverse abdominal muscle^(17)^. This procedure interrupts the sensory innervation to the anterior abdominal wall and peritoneum^(18)^. Some trials have shown that TAP blocks lead to a significant reduction in narcotic consumption and recovery times in both open and laparoscopic surgery^(19)^.

In our meta-analysis, we aimed to estimate the effect of ropivacaine infiltration in the reduction of postoperative pain and the total need for opioids in the first 24 h postoperatively.

This systematic review and meta-analysis were performed with strict adherence to the Preferred Reporting Items for Systematic Reviews and Meta-analyses (PRISMA) Statement^(20)^. In addition, we followed the guidelines reported in the Cochrane Handbook for Systematic Reviews of Interventions^(21)^.

### Literature Search

We searched for published studies in four online databases: PubMed, Web of Science, Scopus, and Cochrane Central Register of Controlled Trials (CENTRAL) in August 2020. Our search was performed using the following keywords: ropivacaine, naropin, laparoscop*, and hysterectom* and combining these words with “AND” or “OR” as was necessary according to the search engine being used.

### Eligibility Criteria

For eligibility, we included all studies that met all of the following criteria: (1) Patients: women undergoing laparoscopic hysterectomy, (2) Intervention: ropivacaine, (3) Comparator: placebo, (4) Outcomes: pain intensity [measured either using visual analog scale (VAS) scores or numerical rating scale (NRS) scores], overall quality of recovery (QoR-40 score), which is measured by physical comfort, physical independence, pain, emotional status, and support, and the need for opioid rescue. Type of Study: we only included randomized clinical trials (RCTs). Studies with other criteria were excluded, including (1) non-RCTs, (2) single-armed trials or with different comparators, (3) trials involving animals, and (4) studies for which there was no availability of a full-text copy of the paper.

### Screening and Studies Selection

Our next step was to export the search results from our databases into Endnote X8.0.1 (Build 1044) and perform automatic removal of any duplicates. Following this, we screened the search results manually in two steps: first, we performed title and abstract screening, then we went on to perform full-text screening for the preliminary studies included in the first step. We included articles based on our criteria for eligibility and removed studies that did not fulfill these criteria.

### Data Extraction and Analysis

After the screening step, we extracted data from the eligible studies. Data extracted were categorized into two main groups: (1) Demographic and baseline data of patients in each study including age, body mass index (BMI), sample size, dose of intervention, surgery time, blood lose, number of patients diagnosed by fibroid, number of patients diagnosed with endometriosis, number of patients diagnosed with prolapse, number of patients diagnosed with chronic pelvic pain. (2) Data for analysis include pain intensity (by VAS or NRS score), quality of recovery (QoR-40) score that includes physical comfort, physical independence, emotional status, pain RoQ40, and support. Additional outcomes included anti-emetic use and need for opioids. Data for continuous outcomes were extracted as a mean and standard deviation, and data for dichotomous outcomes were extracted as events and total.

### Data Analysis

We performed this analysis using the Review Manager software (RevMan 5.3). Data for continuous outcomes are expressed using mean difference (MD) and standard deviations, and dichotomous outcomes are expressed using percentage and total relative to a fixed 95% confidence interval (CI). We used standardized mean difference (SMD) whenever outcomes were measured using different scores. Heterogeneity was assessed using a statistical I^2^ test and p-value of the chi-square test, where outcomes with I^2^ >50%, p<0.1 were considered heterogeneous, and outcomes with I^2^ <50%, p>0.1 were considered homogeneous. Next, homogenous data were analyzed using a fixed-effects model, and the heterogeneous outcomes were analyzed using the random-effects model. In heterogeneous data not solved using the leave-one-out method, we then conducted a subgroup meta-analysis as the next step in attempting to solving heterogeneity.

### Quality Assessment

We performed a quality assessment by an evaluation which used the GRADE Guidelines (Grading of Recommendations Assessment, Development and Evaluation). For our risk of bias (ROB) assessment, we used the Cochrane ROB tool for use in clinical trials^(22)^. The Cochrane ROB assessment tool includes the following domains: random sequence generation (selection bias), allocation sequence concealment (selection bias), blinding of participants and personnel (performance bias), blinding of outcome assessment (detection bias), incomplete outcome data (attrition bias), selective outcome reporting (reporting bias) and other potential sources of bias. The authors’ judgment is categorized as “Low risk”, “High risk” or “Unclear risk” of bias.

### Results of Literature Search

**Figure 1:**
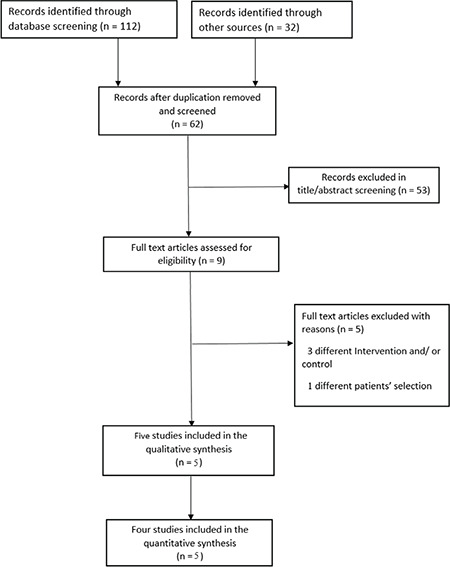
PRISMA flowchart diagram

Out of 144 studies included through our literature search and references, only nine studies were eligible and included in the full-text literature. Five studies fulfilled our eligibility criteria after full-text screening and were included in our meta-analysis. Figure 1 illustrates the PRISMA statement of our literature search.

Three hundred eight patients were included (166 in the ropivacaine group, and 142 in the control group). The mean age of patients in the study group was 50.2±10.9 years, and the mean age of the control group was 51.2±12.8 years. The mean BMIs in the intervention group and the control group were 26.7±5.8 and 27±5.3 kg/m^2^, respectively. Detailed baseline characteristics for the included studies are shown in Table 1. The mean duration of surgery in the ropivacaine group and control group were 147.3 and 138.8 minutes respectively, and the mean blood loss was 92.5 and 72.5 mL, respectively. Table 2 summarizes the surgical duration and blood loss in each study.

### Results of Quality Assessment

**Figure 2:**
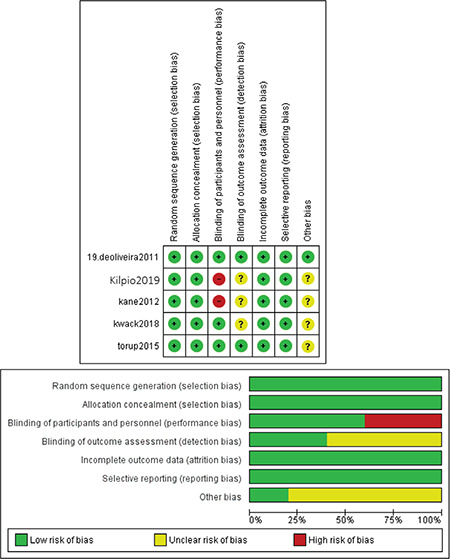
Risk of bias chart

The overall ROB of included studies was of low risk according to the Cochrane ROB assessment tool. All studies were at low risk regarding random sequence generation (selection bias) and allocation concealment. Three studies^(23,24,25)^ performed proper blinding of personnel and participants and therefore were considered as low risk, whereas the other two studies^(26,27)^ were at high risk of performance bias. Outcome assessors were blinded in De Oliveira et al., 2011^(23)^ and Torup et al.^(25)^ 2015 and considered at low risk of detection bias. Three studies did not report whether outcome assessors were blinded and therefore were considered to have unclear risk of detection bias^(24,26,27)^. All studies were of low ROB regarding attrition bias and reporting bias. No other ROB was detected in any study. Figure 2 illustrates the ROB of included studies.

## Results of Outcomes

### Pain Score

**Figure 3:**
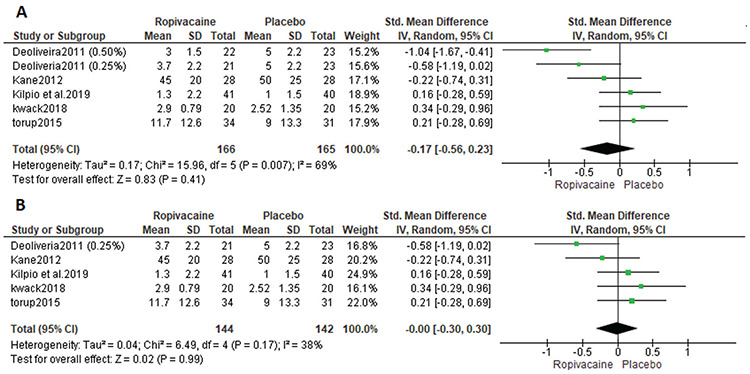
Postoperative pain in included studies SD: Standard deviation, CI: Confidence interval

All included studies reported pain score outcomes. Three studies reported pain score outcomes using VAS scores^(25,26,27)^, whereas the others used NRS scores^(23,24)^. Therefore, we used the SMD. The analysis showed no significant difference between the ropivacaine and placebo groups [SMD=-0.17, 95% CI: (-0.56, 0.23); p=0.41] (Figure 3A). Data were heterogeneous (p=0.007, I^2^=69%). In an attempt to solve the heterogeneity, we excluded one study^(23)^ (0.50% mg) from the analysis. Pooled analysis did not favor any one group over any other [SMD=-0.00, 95% CI: (-0.30, 0.30); p=0.99]. Data were homogeneous (p=.17, I^2^=38%). Figure 3B shows the analysis of pain score outcomes after the leave-one-out method.

### QoR-40 Score

**Figure 4:**
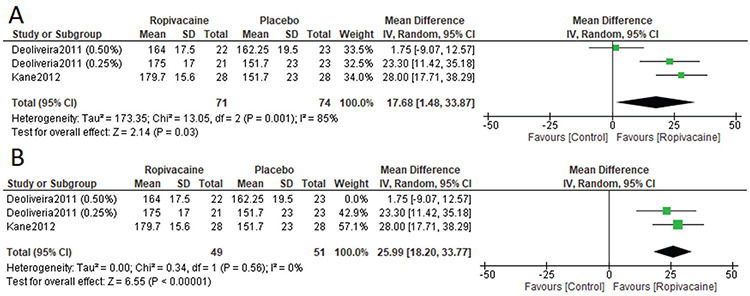
RoQ scores in included studies SD: Standard deviation, CI: Confidence interval

De Oliveria et al.^(23)^ 2011 and Kane et al.^(26)^ 2012 reported QoR-40 score outcomes. The analysis of overall RoQ40 score favored the ropivacaine group over the control group significantly [MD=17.68, 95% CI: (1.48, 33.87); p<0.001]. Data were heterogeneous (p=0.001, I^2^=85%) (Figure 4A). Heterogeneity was best solved by employing the leave-one-out method to exclude De Oliveira et al.^(23)^ 2011 (0.50%) (p=0.56, I^2^=0%), and there was significant favoring of the ropivacaine group over the control group [MD=25.99, 95% CI: (18.20, 33.77); p<0.001] (Figure 4B).

**Figure 5:**
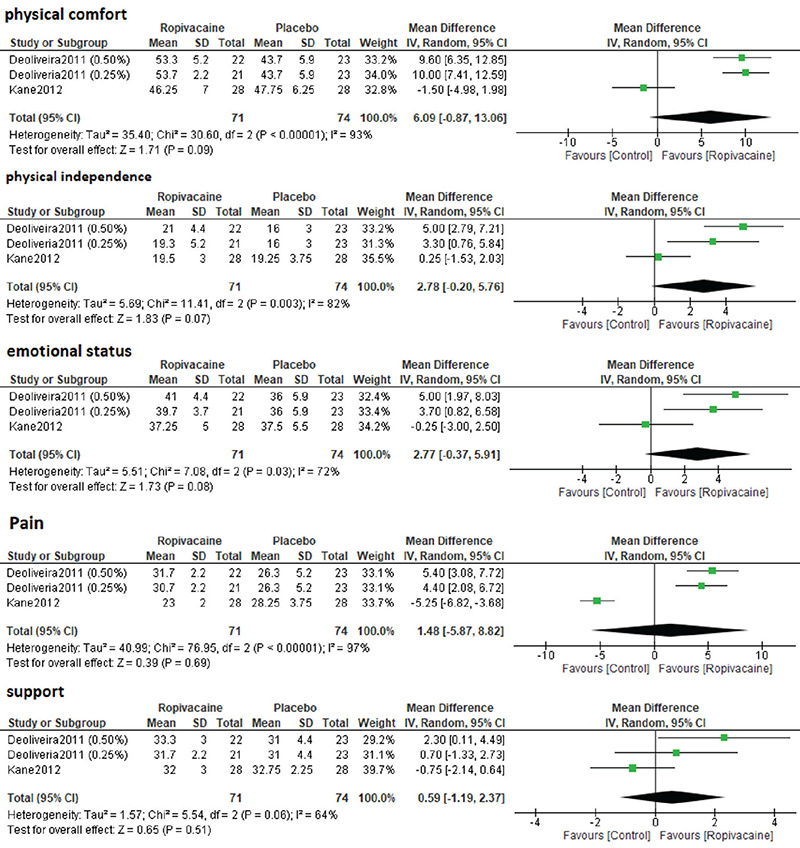
Components of RoQ scores SD: Standard deviation, CI: Confidence interval

Detailed analysis for each item of the QoR-40 score (physical comfort, physical independence, emotional status, pain, and support) is shown in Figure 5; there was no significant difference between the two groups regarding each item of QoR-40.

### Opioid Rescue

**Figure 6:**
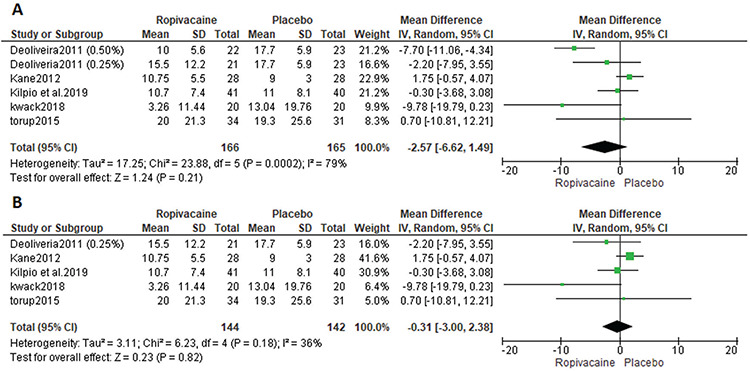
Incidence of use of opioid rescue SD: Standard deviation, CI: Confidence interval

All studies reported opioid rescue outcomes. The analysis  showed no significant difference between the groups [MD=-2.57, 95% CI: (-6.62, 1.49); p=0.21]. Data were heterogeneous (p<0.001, I^2^=79%) (Figure 6A). Heterogeneity was best solved using the leave-one-out method excluding De Oliveira et al.^(23)^ 2011 (0.50% mg) (p=0.18, I^2^=36%). The net result of the analysis showed no significant difference between the groups [MD=-0.31, 95% CI: (-3.00, -2.38); p=0.82]. Figure 6B shows the analysis of opioid rescue outcomes after the leave-one-out method.

## Discussion

Our analysis found that the ropivacaine neither significantly reduce pain following laparoscopic hysterectomy nor opioid consumption in the first 24 h. It significantly controlled overall RoQ40, but there was no difference between ropivacaine and placebo in control items of the QoR-40 score (physical comfort, physical independence, emotional status, pain, and support). Of the five included studies, three administered ropivacaine through (TAP)^(23,25,26)^, one through vaginal cuff infiltration^(27)^, and one simply administered it vaginally^(24)^. Kwack et al.^(24)^ assessed pain score at different hours during the entire 24 hours and found that ropivacaine was superior to placebo in reducing postoperative pain intensity only at 2 h, but there was no significant difference in pain reduction at 6, 12, and 24 h. De Oliveira et al.^(23)^ compared two different concentrations of ropivacaine (0.5% and 0.25%) with saline, concluding that there was no difference between the 0.25% ropivacaine group or the 0.5% ropivacaine group and the saline group in the reduction of postoperative opioid consumption^(23)^.

The use of the TAP block in most of our studies could explain why ropivacaine did not significantly control post-laparoscopic hysterectomy pain, which usually arises from the perineum, shoulder, and abdomen. Abdominal pain originates from somatic and visceral components with the visceral pain being stronger^(28)^. A TAP block potentially covers somatic pain only because it blocks sensory nerves in the thoracolumbar region that supply the anterolateral abdominal wall^(29,30,31)^. Some authors theorized that for the reduction of postoperative pain, ropivacaine should be administered in such a way as to be absorbed systemically^(32)^. A review by Shin et al.^(33)^ demonstrated that TAP block was not significant in pain reduction and morphine consumption in the first 24 h following laparoscopic hysterectomy. Kwack et al.^(24)^ focused on control visceral pain through the injection of ropivacaine into the uterosacral area to block pelvic visceral plexus (uterine nerve plexus). They found that there was a reduction in early postoperative pain and the need for analgesics^(33)^.

Acharya et al.^(34)^ and Chiruvella et al.^(35)^ concluded that adding dexmedetomidine to ropivacaine was effective in the management of postoperative pain and reduced analgesic consumption following laparoscopic hysterectomy. They also found that this combination was superior to using ropivacaine alone^(34,35)^. In comparison with lidocaine, Ghisi et al.^(36)^ found no difference in the analgesic effect of ropivacaine in pain control after laparoscopic abdominal surgeries, but the cost of lidocaine was lower than that of ropivacaine.

Chou et al.^(28)^ showed that the use of ropivacaine via the intraperitoneal route was effective in pain control and reduced analgesic consumption after laparoscopic appendectomy. Thakur et al.^(37)^ found that the ropivacaine significantly reduced postoperative pain and analgesic consumption following laparoscopic cholecystectomy through combined wound and intraperitoneal instillation. Likewise, Yong and Guang^(38)^ found that ropivacaine could reduce pain following laparoscopic cholecystectomy, but only when administered through an intraperitoneal installation^(39)^.

A RCT by Korkmaz et al.^(39)^ showed that bupivacaine could significantly reduce VAS scores and tramadol consumption after laparoscopic hysterectomy^(40)^. However, Chatrath et al.^(41)^ found that ropivacaine was better than bupivacaine in its analgesic effect with fewer adverse effects.

The main strength of our analysis was that it included only RCTs with low ROB. The main limitation of our study was the very low number of studies, the fact that we were forced to pool studies in which ropivacaine was injected as part of a TAP block along with those that administered or injected vaginal ropivacaine.  More clinical trials are needed to investigate the efficacy of ropivacaine in pain relief after laparoscopic hysterectomy, and ultimately studies need to be performed to differentiate the efficacy and advantages of the different routes of administration. In conclusion, our analysis found that ropivacaine did not significantly reduce pain intensity and analgesic consumption after laparoscopic hysterectomy.

## Figures and Tables

**Table 1:**
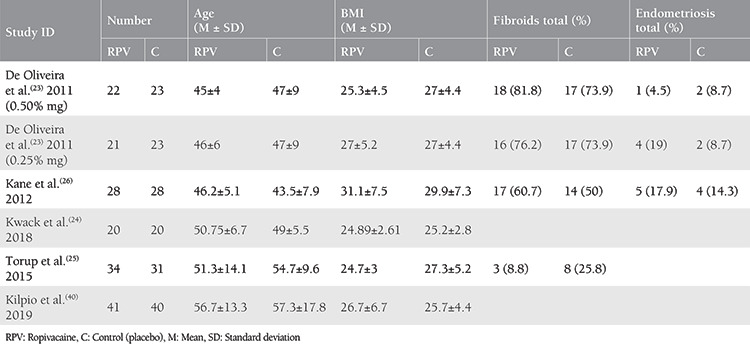
Baseline characteristics of the included studies

**Table 2:**
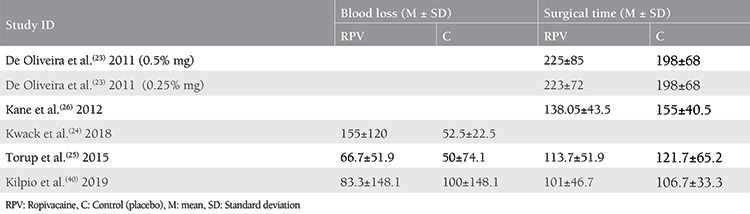
Surgery time and blood loss estimation in each of the included studies
